# An assessment of the contribution of National Stop Transmission of Polio Program to Nigeria’s Immunization Program

**DOI:** 10.11604/pamj.supp.2021.40.1.15816

**Published:** 2021-11-11

**Authors:** Oladayo Biya, Wiedad Roodly Archer, Julia Rayner, Ralph Welwean, Ayodele Jegede, Sara Jacenko, Sarah Pallas, Taiwo Abimbola, Kirsten Ward, Eric Wiesen

**Affiliations:** 1 Polio Eradication Branch, Global Immunization Division, Centers for Disease Control and Prevention, Atlanta, United States of America,; 2 Control of Infectious Diseases, London School of Hygiene and Tropical Medicine, London, England,; 3 Department of Sociology, University of Ibadan, Nigeria,; 4 Global Immunization Division, Centers for Disease Control and Prevention, Atlanta, United States of America

**Keywords:** National Stop Transmission of Polio (NSTOP) program, poliovirus, routine immunization, Government of Nigeria, program evaluation

## Abstract

**Introduction:**

In July 2012, the National Stop Transmission of Polio (NSTOP) program was established to support the Government of Nigeria in interrupting transmission of poliovirus and strengthen routine immunization (RI). NSTOP has approximately 300 staff members with the majority based at the Local Government Area (LGA) level in northern Nigeria.

**Methods:**

An internal assessment of NSTOP was conducted from November 2015 to February 2016 to document the program´s contribution to Nigeria´s immunization program and plan future NSTOP engagement. A mixed methods design was used, with data gathered from health facility, LGA, state, and national levels, through structured surveys, interviews, focus group discussions, and review of program records. Survey and expenditure data were summarized by frequency and trends over time, while interview and focus group data were analyzed qualitatively for key themes.

**Results:**

The majority of the 111 non-NSTOP LGA respondents reported that NSTOP officers supported polio campaigns (100%) and supervised RI sessions (99.1%). Out of 181 respondents at health facility level, the majority reported that NSTOP trainings improved their knowledge (83.3%) and skills (76.2%) on RI, and NSTOP officers regularly supervised their RI sessions (96.7%). Most respondents reported that there would be a negative impact on immunization activities if NSTOP officers were withdrawn.

**Conclusion:**

Future implementation of NSTOP should be realigned to (a) give highest priority to mentoring LGA staff to build institutional capacity, (b) ensure increased capacity translates to improved provision of RI services, and (c) improve routine review of program monitoring data to assess progress in both polio and RI programs.

## Introduction

In July 2012, the National Stop Transmission of Polio (NSTOP) program was established in Nigeria to support the Government of Nigeria in interrupting transmission of poliovirus and strengthen Routine Immunization (RI). This was based on the need identified in the 2012 National Polio Eradication Emergency Plan (NPEEP) for the health workforce to have management and technical surge capacity to strengthen the Nigerian polio program [1]. NSTOP was created as a collaborative effort of the US Centers for Disease Control and Prevention (CDC), the Nigeria National Primary Health Care Development Agency (NPHCDA), and the Nigeria Field Epidemiology and Laboratory Training Program (NFELTP) [2]. NSTOP is modeled after the CDC-World Health Organization (WHO) STOP program [3] but is staffed by Nigerian citizens. The key intended outcomes of the program are improved planning and implementation of Supplemental Immunization Activities (SIAs), improved knowledge and skills of trained government staff, greater uptake of RI vaccines, better management of RI data, improved detection and interruption of Transmission of Wild Poliovirus (WPV) and circulating Vaccine-Derived Poliovirus (cVDPV), and effective response to polio and other Vaccine-Preventable Diseases (VPD) outbreaks.

The flagship project of NSTOP was an initiative to locate and vaccinate children < 5 years with Oral Poliovirus Vaccine (OPV) in remote Fulani, nomadic, scattered, and border populations in northern Nigeria. From August 2012 - April 2015, NSTOP conducted field outreach activities in Northern Nigeria where 64,131 children aged < 5 years, who had never received polio vaccination, were vaccinated with OPV [4]. Beyond the flagship project, NSTOP has provided additional support to the polio and RI programs in Nigeria. Since 2012, NSTOP has deployed Management Support Teams (MSTs) to polio high-risk Local Government Areas (LGAs) to improve polio campaign performance. In 2013, NSTOP started recruiting officers for polio high-risk LGAs in 12 northern states and Federal Capital City (FCT) to provide technical and management support to the LGA team on Polio Eradication Initiative (PEI) and RI. The NSTOP LGA officers (NSLOs) were recruited in a gradual manner based on the needs of the country over a one-year period and were given specific Terms of Reference (TORs) to support different components of the polio program such as polio campaigns, surveillance and outbreak investigation, and RI strengthening. From 2013 to 2015, group trainings on nine RI thematic areas were conducted at regular intervals for state, LGA, and health facility immunization officers in the 12 states and FCT. In 2014, NSTOP in collaboration with National Primary Health Care Development Agency (NPHCDA) piloted the District Health Information System version 2 (DHIS-2) and RI dashboard in Kano state with the aim of improving RI data management and use [5]. The project is presently being scaled up to cover all states in Nigeria. In 2015, CDC and NSTOP initiated a project to support states and LGAs to achieve certification-level standard for Acute Flaccid Paralysis (AFP) surveillance. In November 2015 and January 2016, NSTOP supported the conduct of measles SIAs in northern and southern parts of Nigeria respectively.

NSTOP works closely with government and partners to achieve its mission of polio eradication and RI strengthening. NSTOP stakeholders include government agencies and departments at national, state, and LGA levels, and partner organizations such as WHO, UNICEF, Bill and Melinda Gates Foundation (BMGF), United States Agency for International Development (USAID), and African Field Epidemiology Network (AFENET). CDC, USAID, and BMGF fund NSTOP. NSTOP has approximately 300 staff, consisting of a National Coordinator (NC), a deputy NC, five Abuja-based Field Coordinators, 19 State Field Coordinators (SFCs) across 11 states, 19 DHIS-2 Implementation Officers, and 216 NSTOP LGA officers (NSLOs). The number of State Field Coordinators (SFCs) in a state ranges from one in most states to four in Kano state. There is usually one NSLO per LGA except for LGAs that receive concurrent polio/RI and malaria support, which have two NSLOs.

On the current monitoring system in NSTOP, a calendar of program activities is prepared at the beginning of the year and is updated regularly. The calendar includes NSTOP internal program activities and other activities initiated by government and partners that NSTOP participates in, with the dates of the activities. At the LGA level, NSLOs prepare monthly updates to itemize key activities performed in the previous month. This is sent to the SFC, who reviews and gives feedback to the NSLOs at a joint meeting held at the end of the month. The SFC prepares a monthly summary update that is sent to the national office. At the national level, a monthly summary of key activities is written and included as part of NFELTP monthly update. In order to have in-depth understanding of state and LGA-level activities, a quarterly NSTOP review meeting is held, comprising national and state NSTOP officers.

Since NSTOP program´s inception in 2012, no formal evaluation had been conducted. In 2015, an internal assessment of NSTOP was conducted to: a) describe the process of implementation of NSTOP; b) identify strengths, challenges and ways the program could be improved; c) document the contributions of the program to the polio and RI programs in Nigeria, all of which would help inform planning for future implementation of NSTOP. The assessment was designed to gain a deeper understanding of the program´s strengths and challenges rather than quantify the impact of the program, as the many other investments in improving immunization during the same time period make it difficult to tease out the impact of any one initiative. In this paper, we report the findings of the internal assessment of NSTOP conducted by CDC.

## Methods

The internal assessment of NSTOP was conducted between November 2015 and February 2016. The assessment focused on implementation of NSTOP, following CDC´s guidelines for evaluation of public health programs [6]. A mixed methods design was used [7]. The quantitative component included surveys and a structured review of program documents. The qualitative component included in-depth interviews and focus group discussions.

### Quantitative analysis

#### Survey of immunization officers

**Figure 1:**
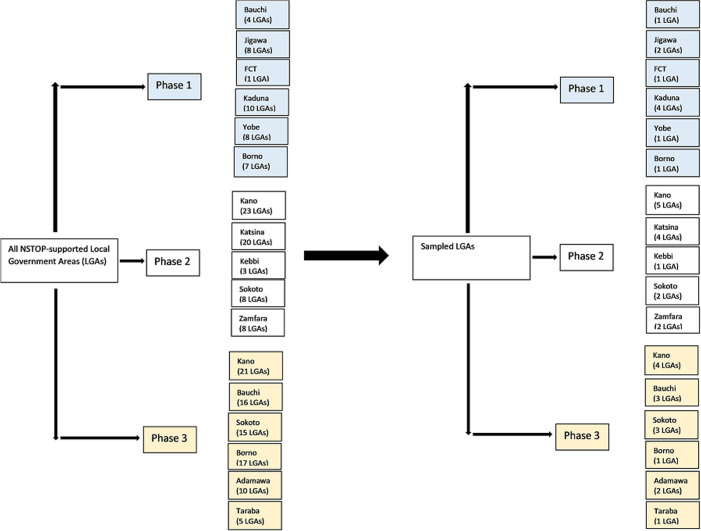
Location of all NSTOP-supported LGAs in Nigeria and those LGAs sampled for the internal assessment of NSTOP

We conducted a cross-sectional survey of key immunization technical officers at state, LGA, and Health Facility (HF) levels in 12 NSTOP-supported states and FCT. At the state level, we surveyed 13 State Immunization Officers (SIO). To select LGAs, we stratified the 184 NSTOP-supported LGAs by the three phases of recruitment (April 2013, July 2013, June 2014). We then used simple random sampling proportional to the number of NSTOP-supported LGAs in a state to sample 38 LGAs (Figure 1). We sampled randomly using computer-generated numbers, where all NSTOP-supported LGAs were listed by state, and a state with a higher number of LGAs will have more LGAs selected. All security-compromised LGAs in Borno, Yobe, and Kaduna states were excluded due to challenges in accessing these areas. We also used simple random sampling with computer-generated numbers to select three HFs in each of the 38 selected LGAs, 152 LGA officers and 228 health facility officers were sampled purposively (Table 1). For both state and LGA surveys, we used a pretested semi-structured questionnaire to collect data on activities of NSTOP officers, impact of NSTOP support, and likely effects of withdrawal of NSTOP officers. All survey data were cleaned and analyzed using SAS 9.3 software. We determined frequencies and proportions of variables.

**Table 1:** sample size for cross sectional survey

Level	Sample size	Method	Respondents
State	13	All 12 states and Federal Capital Territory (FCT)	State Immunization Officers
Local Government Area (LGA)	152	38 LGAs sampled from 12 States and FCT 114 Non-*NSTOP LGA officers, 3/LGA 38 NSTOP LGA officers, 1/LGA	WHO LGA Facilitator, UNICEF Consultant, Local Immunization Officer NSTOP LGA officer
Health facility (HF)	228	114 HFs sampled from 38 LGAs 2 HF officers/LGA	HF-in-charge and Routine Immunization focal person

* NSTOP - National Stop Transmission of Polio

#### Review of program records and expenditure analysis

From November 2015 to February 2016, we collated and reviewed two categories of NSTOP program data that had been collected prior to the commencement of the assessment: (a) RI baseline and follow-up assessment data for the first two phases of NSTOP recruitment. These data included interviews from LGA and HF immunization officers. Of the 100 phase 1 and phase 2 LGAs, only 89 had data collected. Of these 89 LGAs, only 77 had complete data at both baseline and follow-up and were included in the analysis. Data were analyzed at LGA and HF levels to measure changes in RI performance. Paired T-tests, with pairs of the same LGA at baseline and follow-up, were used to compare differences in continuous variables, and McNemar´s chi-squared tests used to compare changes in binary variables using Stata 14.0, with p values of <0.05 treated as statistically significant. (b) NSTOP program expenditure data from the United States Fiscal Years (FY) 2012 to 2015 reported in constant 2015 U.S. dollars. A FY is from October through September of the following year. Program expenditure data were reported by NSTOP program administrators for all funding sources (CDC, USAID, and BMGF) supporting the program for FY2012-FY2015. Program expenditure data were analyzed in Microsoft Excel.

### Qualitative analysis

#### Interviews with key stakeholders

We conducted in-depth interviews with key national, state, and LGA stakeholders in the NSTOP program. We obtained information from several stakeholders at different levels who had contact with NSTOP´s operations and management, in order to make a good judgement of NSTOP´s contribution. At the national level, we interviewed 17 senior immunization program officers sampled purposively from NPHCDA (including the national polio Emergency Operations Center (EOC), WHO, UNICEF, USAID, AFENET, and CDC Nigeria). At the state level, we interviewed 78 senior immunization program officers sampled purposively from State Primary Health Care Development Agency (SPHCDA), WHO, UNICEF, and AFENET from all 12 NSTOP-supported states and FCT. In each of the 38 sampled LGAs, we interviewed the Director of Primary Health Care (PHC). At all the levels, we used a pre-tested interview guide to collect data on activities of NSTOP officers, perceived impact of NSTOP support, and likely effects of withdrawal of NSTOP officers.

#### Focus group discussions with NSLOs

Using a pre-tested guide, seven focus group discussions (FGDs) were conducted with NSLOs to collect data on their experience with supporting immunization activities in the LGAs. One FGD was conducted in each of the following NSTOP-supported states: Kaduna, Jigawa, Zamfara, Kano, Katsina, Sokoto, and Bauchi. Each FGD included 8-10 NSLOs sampled purposively from the pool of NSLOs in those states. The main criterion used for sampling was length of experience. Coding and thematic analysis of all qualitative data were done using ATLAS.ti 7.

### Indicators

From a logic model we developed for NSTOP, we identified key program outcome indicators to use in assessing NSTOP program performance. These included (a) change in knowledge and skills of immunization staff, (b) changes in planning and conduct of RI, (c) changes in planning and conduct of SIAs, and (d) vaccination coverage for Oral Polio Vaccine (OPV) delivered through polio campaigns in NSTOP-supported LGAs. Additional output indicators were used to assess extent of mentorship of immunization officers at state and LGA levels by NSTOP staff use of NSTOP program funds.

### Human subject determination

The national polio Emergency Operations Center (EOC) approved the assessment as a non-research programmatic activity. All participants were provided information about the assessment verbally and requested to provide written consent prior to participation.

## Results

For the survey, there were 181 respondents across 114 HFs, out of which 103 (56.9%) were HF in-charge and 78 (43.1%) RI focal persons. At the LGA level, there were 148 respondents in all, comprising 111 non-NSTOP respondents and 37 NSTOP respondents. Out of the 111 non-NSTOP respondents, 39 (35.1%) were WHO LGA facilitators, 37 (33.3%) LGA immunization officers (LIOs) and 35 (31.5%) UNICEF consultants. All the 37 NSTOP respondents were NSLOs. At the state level, there were 13 respondents, all SIOs. We analyzed baseline and follow-up data for 77 LGAs and 1401 HFs. For FGDs and in-depth interviews, there were 66 and 133 respondents respectively.

### Quantitative analysis

#### Survey of immunization officers

##### Effect of NSTOP program

**Figure 2:**
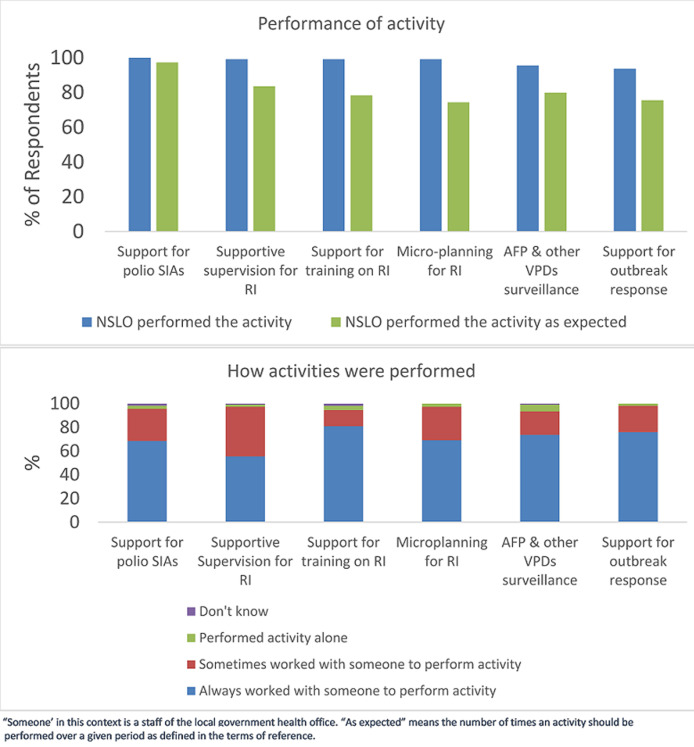
Assessment of performance of NSTOP officers by their peers at the local government level (N=111)

Figure 2 shows non-NSTOP LGA officers views on the frequency at which NSLOs performed key activities and how often these were performed in collaboration with LGA staff. Of surveyed officers, 81% reported that NSLOs always worked with someone (another LGA staff), as part of capacity transfer, to provide training on RI and 55% indicated that NSLOs always worked with someone to provide supportive supervision for RI.

Table 2 illustrates the perceived effect of NSTOP activities and the likely effect of withdrawing NSTOP officers from non-NSTOP LGA officers and state respondents. A minority of respondents (8% at LGA and 15% at state) strongly agreed that the government at their levels was prepared to take over NSTOP responsibilities. A majority reported that there would be a negative effect on all activities indicated from withdrawing NSTOP officers, as compared to no effect or a positive effect. A negative effect in this regard refers to a slowing down or an outright failure of the PEI process.

**Table 2:** perceived effect of NSTOP activities given by non-NSTOP LGA officers during survey (N=111)

		LGA (%)						State (%)					
Theme	Sub-theme	Strongly agree	Agree	Neutral	Disagree		Strongly disagree	Strongly agree	Agree	Neutral	Disagree		Strongly disagree
Perceived effect of NSTOP activities	NSTOP participation has increased effectiveness of the team	67.89	25.69	2.75	0		3.67	69.23	15.38	0	7.69		7.69
	NSTOP effectively coordinates its activities with the team	73.64	22.73	0	0.91		2.73	69.23	23.08	0	7.69		0
	NSTOP is effective at the health facility and LGA levels	69.09	22.73	1.82	2.73		3.64	61.54	30.77	0	0		7.69
	You are prepared to take over NSTOP responsibilities at this level	8.11	8.11	18.92	23.42		41.44	15.38	23.08	30.77	15.38		15.38
		LGA (%)						State (%)					
Theme	Sub-theme	Negative effect	No effect			Positive effect		Negative effect		No effect		Positive effect	
Likely effect of withdrawing NSTOP officers on specific activities	Supportive supervision for RI	86.49	0.9			12.61		76.92		0		23.08	
	Support for polio SIAs	85.59	1.8			12.61		76.92		0		23.08	
	Support for training on RI	85.59	1.8			12.61		76.92		0		23.08	
	Microplanning for RI	85.32	1.83			12.84		83.33		0		16.67	
	Data management for RI and IPDs	83.64	3.64			12.73		75		8.33		16.67	
	Support for training on polio SIAs	81.82	5.45			12.73		53.85		23.08		23.08	
	Vaccine and cold chain management	81.08	6.31			12.61		Not applicable		Not applicable		Not applicable	
	AFP and other VPDs surveillance	80.18	7.21			12.61		75		8.33		16.67	
	Social mobilization	78.38	9.01			12.61		Not applicable		Not applicable		Not applicable	
	Support for outbreak response	76.36	10.91			12.73		75		8.33		16.67	

At the HF level, a majority of respondents (97%) reported that NSTOP officer had supervised at least one RI session, that they had attended NSTOP training (93%), and that the trainings improved their knowledge (83%) and skills (76%) on RI. From NSTOP trainings, the training topic the HF officers remembered most frequently was RI service delivery (49%) and some of the terms respondents used to recall this topic included vaccine introduction, vaccine training, routine immunization and supportive supervision. This topic was followed by vaccine and cold chain management (26%), and some of the terms used to recall the topic include temperature monitoring and vaccine supply.

##### Barriers to NSTOP implementation

According to NSLOs, the most common factor hindering them from performing their job was lack of security (42%), particularly in the underserved settlements and hard to reach areas in northern Nigeria. NSLOs also reported broader barriers effecting the polio eradication initiative (PEI) in their LGAs which also effected their work. Lack of government ownership (73%), low motivation and commitment among health workers (57%), and poor management of resources (51%) were reported to be the biggest obstacles.

##### Additional assistance required for improved performance

When HF officers were asked what additional assistance they would need to maximize their performance, the most commonly mentioned were human resources (38%), logistic (34%) and material incentives (15%). The human resource needs to include request for more RI personnel and refresher trainings. Logistic needs included requests for motorcycles to visit hard-to-reach areas and cold chain equipment for transport of vaccines and related supplies during outreach. NSLOs offered suggestions on methods, in addition to training, to prepare LGA immunization teams for their roles. A majority of NSLOs suggested promotion opportunities (87%) and rewards for good performance (60%) as additional strategies.

##### Recommendations to improve NSTOP program

Health facility officers were asked how NSTOP could improve its trainings. The main recommendations were to increase the frequency of training to about monthly from a need-based approach (35%) and expand the target audience to include other staff members (28%). To improve NSTOP program overall, non-NSTOP LGA officers (32%) and SIOs (50%) recommended that NSTOP should increase human resources such as increase the number of its staff, followed by a recommendation for NSTOP to organize refresher trainings. SIOs (14%) also recommended that logistics for supportive supervision should be provided.

#### Review of records and expenditure analysis

##### Results from the RI baseline and follow-up assessment

Table 3 illustrates the change in key variables between baseline and follow up. The mean percentage of staff trained increased by 27% (p<0.01) at LGA level and by 25% (p<0.01) at the health facility level. The mean percentage of HFs with a current micro-plan available increased by 20% (p<0.01) but no statistically significant change was seen at the LGA level. At the HF level, the mean percentage reporting vaccine stock-out as a reason for not conducting vaccination sessions decreased by an average of 7% (p<0.05). Stock-outs significantly decreased for measles and yellow fever vaccines, and diluents at both LGA and HF levels. Staff shortages and competing priorities significantly decreased by 21% and 20% respectively (p<0.01 for both). The percentage of LGAs using a checklist for RI supportive supervision visits increased by 17% (p<0.05).

**Table 3:** baseline and follow up indicators on routine immunization at local government and health facility levels (N=77 LGAs)

	LGA DATA					HF DATA				
Training	BL	FU	Diff.	P-Value	Obs	BL	FU	Diff.	P-Value	Obs
Percentage of government staff trained‡	59.1	86.4	27.30	<0.01*	74	50.1	75.4	25.28	<0.01*	75
Microplanning										
Presence of Microplan	89.3	92.4	3.03	0.77	66	58.8	78.8	20.00	<0.01*	75
Proportion of LGAs with microplans that contain:										
ID of hard-to-reach populations	98.1	86.8	-11.32	0.03*	53	60.3	74.3	14.00	<0.01*	69
Outreach plan	70.6	100	29.41	<0.01*	51	75.3	79.2	3.90	0.37	69
Percentage of HFs yet to receive RI services in 3 months due to:										
Vaccine stockout	33.8	19.5	-14.29	0.06	77	8.27	0.9	-7.36	<0.01*	62
Staff shortages	26	9.1	-16.88	0.01*	77	23.4	3	-20.47	<0.01*	62
Competing priorities	31.2	13	-18.18	0.01*	77	21.7	1.7	-20.03	<0.01*	62
Insufficient funding	28.6	19.5	-9.09	0.26	77	23.6	2.14	-21.44	<0.01*	62
Insufficient transport	28.6	15.6	-12.99	0.09	77	23	2.2	-20.76	<0.01*	62
Insecurity or violence	16.9	24.7	7.79	0.24	77	6.8	1.2	-5.58	0.05	62
Stock-outs										
tOPV						13.9	5.8	-8.13	<0.01*	76
Measles	31.5	11	-20.55	<0.01*	73	24.7	11.87	-12.69	<0.01*	75
BCG	25	12.5	-12.5	0.06	72	38.4	21.9	-16.52	<0.01*	76
DPT	9.7	5.6	-4.17	0.51	72	13.4	7.6	-5.84	0.05	76
Yellow fever	34.2	15.1	-19.18	0.02*	73	23.9	11.9	-12.03	<0.01*	75
Individual vaccination record cards	37	23.3	-13.7	0.06	73	22.6	12	-10.68	<0.01*	75
Cold Chain										
Working thermometer	86.5	82.4	-4.05	0.65	74	19.3	29.6	10.31	0.03*	71
Supervision by state officers										
Supervisory checklist used	57.4	74.5	17.02	0.04*	47	26.2	34.2	8.02	0.14	73
Training for HF staff	41.9	71	29.03	<0.01*	62	41.8	54.6	12.73	<0.01*	76
Monitoring										
Report feedback received						41.2	58.9	17.67	<0.01*	76
RI monitoring chart present						67	86.6	19.62	<0.01*	73
RI monitoring charts up-to-date for current month						66.4	79	12.66	<0.01*	71

BL (baseline), FU (follow-up), Diff (difference), Obs (observations), ID (identification). Unless specified, all data are proportions of LGAs that had characteristic ‘x’ at BL or FU; Difference is a percentage difference; values are obtained from McNemar’s -χ2 tests for proportions, with McNemar’s Exact Test P values used. If variables are continuous, matched t-tests were used to obtain mean values at BL and FU and p values. Obs (observations) Number of matching pairs used for statistical test; mean percentages shown calculated with this denominator. ‡This is a continuous variable. * implies statistical significance at p < 0.05 level

##### Expenditure analysis

**Figure 3:**
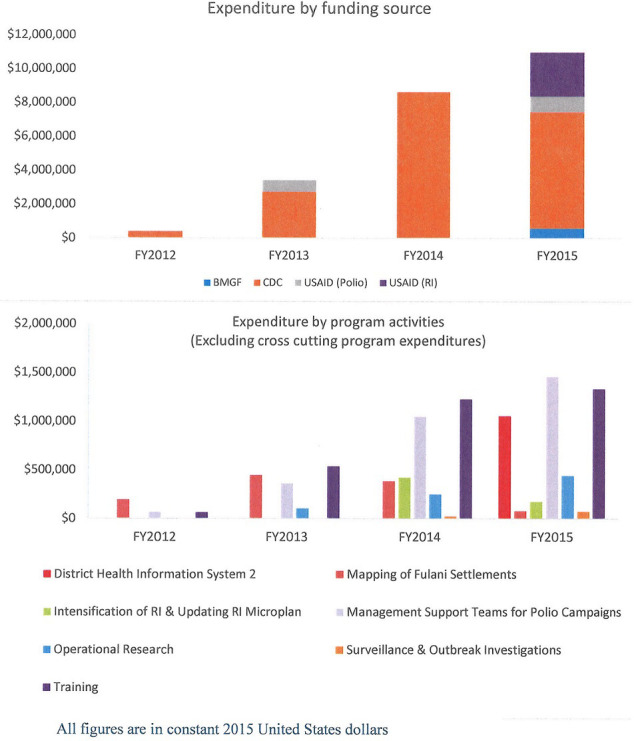
NSTOP program expenditure by funding source and program activities, 2012 - 2015

The total NSTOP program expenditures from FY 2012 to FY 2015 was $28.8 million in constant 2015 US dollars. The top three program area expenditures were cross-cutting program management, which includes personnel salaries ($13.3 million), training ($3.2 million), and MSTs for polio campaigns ($3.0 million). Program expenditures increased yearly from $0.4 million in FY 2012 to $3.5 million (775%) in FY 2013, $8.7 million (149%, compared to 2013) in FY 2014 and $11.0 million (26%, compared to 2014) in FY 2015. CDC was the sole funder in FY 2012 and FY 2014 and was the largest funder in FY 2013 ($2.7 million) and FY 2015 ($6.9 million). Figure 3 shows NSTOP program expenditures by FY and program area but excluding the cross-cutting program management. There was a yearly increase in training expenditures from $0.07 million in FY 2012 to $1.3 million (1,757%) in FY 2015. Similarly, there was a yearly increase in expenditure for MSTs from $0.07 million in FY 2012 to $1.5 million (2,043%) in FY 2015.

### Qualitative analysis

#### Interviews with key stakeholders

On activities of NSTOP officers, there was consensus among stakeholders that NSTOP officers carried out their activities among Fulani and other hard-to-reach communities. A state-level respondent said:

*“The (NSTOP) contributed in reaching the nomadic people. They did a great deal of work in identifying those areas where you have nomadic groups to integrate them and reach them, so they have contributed as part of the team at LGA and at the state level”*.

A majority of respondents also felt that NSTOP officers improved quality of delivery of RI services, especially through supportive supervision in Fulani and other hard to reach areas. According to a LGA-level respondent:

*“I used to see the NSTOP officer dropping his car here and carry kabukabu (motorcycle) and go where motor (car) cannot go and sees that he performed the outreach services”*.

Other activities respondents believed that NSTOP officers carried out include training LGA immunization staff on different RI topics, and participation in microplanning and polio campaign implementation in Fulani and other underserved areas. Few respondents mentioned that NSTOP officers tend to conduct supervision alone, rather than in collaboration with government officers.

Regarding NSTOP support at the national level, a majority believed that NSTOP used the platform of the national polio EOC to work with other partners, and assisted PEI efforts through deployment of senior supervisors (“MSTs) to polio high-risk LGAs. Some other fora of active NSTOP involvement according to respondents include the RI Working Group and Interagency Coordination Committee. Individual opinions about NSTOP at this level include use of phrases such as “NSTOP activities have little planning time”, “NSTOP officers are sometimes not involved in the planning stage of activities”, and “NSTOP officers are fault-finders”.

As training is a major component of NSTOP support, some terms that respondents used to describe NSTOP trainings include “quality”, “relevant”, and “effective”. A national-level respondent said:

*“Yes, I have participated in some of the training at the national and some at state levels, at the operational level, during IPDs (polio campaigns). Yes, I have participated. It has been of quality, it is the outcome of those trainings that you are seeing in terms of accountability, in terms of the dashboard before each campaign”*.

Respondents also believed the trainings contributed to improvement in microplanning and materials from NSTOP training modules helped to train their staff.

Similarly, respondents gave opinions on the overall impact of NSTOP support, weaknesses in NSTOP program, and recommendations to improve NSTOP. A majority believed that NSTOP has positively affected Nigeria´s immunization program. The most common weakness that was mentioned is that NSTOP had insufficient staff. By this, respondents meant that NSTOP should have additional officers to work in more hard-to-reach areas. Individual opinions on the weaknesses are that “NSTOP staff has poor capacity in outbreak response and surveillance” and “NSTOP tends to form parallel structures, rather than complement existing structures”. The recommendations given were to address the weaknesses described above.

### Focus group discussions with NSLOs

The greatest fear of NSLOs was that the impact of NSTOP support might not continue after their withdrawal. The reasons they cited for this include “poor ownership from LGAs´, and “nobody will focus on Fulani and other hard to reach communities”. The NSLOs were generally satisfied with their work. However, the major challenges they faced were lack of access to RI supportive supervision database and lack of funding for outreach vaccination sessions. Other challenges include inadequate cold chain facilities for outreach vaccination sessions and a weak system for holding health workers accountable.

## Discussion

Nigeria´s NSTOP program has provided intensive support for polio campaigns and RI sessions in 184 LGAs with a focus on hard to reach and underserved populations. Across all the levels (LGA, state, and national) where NSTOP officers worked, there was agreement that the NSTOP program has positively influenced the PEI and RI programs in Nigeria. At the state and national levels, respondents indicated that NSTOP officers contributed meaningfully to management decisions, especially at the EOCs and had productive collaboration with other stakeholders. NSTOP had a more pronounced effect at the LGA level where field activities take place. Before NSTOP initiated the flagship project, it was believed that low vaccination coverage in the underserved populations contributed to persistent WPV transmission in Nigeria. Many respondents agreed that NSTOP assisted in improving the quality of polio campaigns in these underserved settlements through improved microplanning and supervision. Respondents at the LGA level believed that the NSLOs worked in line with their TORs. The assessment results indicate that NSTOP support to the polio program at the LGA level is perceived as an integral component of the support provided by other partners and the government. In 2012, it was evident that PEI Nigeria needed all the support it could get from partners to stop the increase in WPV cases. In response to this need, partners mobilized human and financial resources to support several LGAs across northern Nigeria, and NSTOP support was provided within this platform.

Another major program activity for which NSTOP was widely commended is in increasing knowledge of health workers on polio and RI. From 2013 to 2015, there was significant financial investment in training by NSTOP staff which provided the opportunity to train entire LGA immunization and surveillance teams along with core HF officers in LGAs where NSLOs worked. Respondents at LGA and HF levels believed that NSTOP training improved their knowledge and skills and were well organized. The knowledge gained through NSTOP-supported training was felt to have contributed to the observed improvement in some RI indicators which related directly to the training topics. These included improvement in microplan availability, staff availability for RI sessions, monitoring and supervision, and reduction in stock outs of vaccines. For example, NSTOP had a training module specific to microplanning and required NSLOs to assist their LGAs to update their microplans quarterly. NSTOP also provided funding for LGAs to hold a meeting to review microplans. Similarly, as part of their TORs, NSLOs are required to conduct a minimum of eight supportive supervisory visits to health facilities holding RI sessions. During these visits, on-the-job training is performed and observed weaknesses are identified for correction. This was felt to have contributed to the improvement in some monitoring and supervision indicators. The decrease in stock out of some vaccines, on the other hand, may not be fully explained by NSTOP training due to the influence of factors outside the control of those trained, such as availability of vaccines globally and nationally.

Many respondents indicated that government was not ready to take over NSTOP responsibilities and that withdrawal of NSTOP officers would have a negative effect on several activities. A negative effect in this instance could mean a slowing down or an outright failure of the polio eradication program. At the beginning of the NSTOP program, the goal of government and partners was to ensure that polio was eradicated using all available human, financial, and material resources. Although the duration of NSTOP support was not specified at the beginning of the program, it was meant to be time-limited, with functions transitioned to the government as significant progress was made in the PEI. According to NSLOs, lack of government ownership was the greatest threat to the PEI program. This has been documented in other studies on sustainability of immunization program in Nigeria [8,9]. Nigeria´s PEI program is heavily dependent on external partner support. Thus, the lack of government ownership of immunization activities is not just a problem for NSTOP but for all partner organizations in Nigeria. With NSTOP officers handling critical responsibilities on training, supervision and program management, often working in hard-to-reach areas, it is not surprising that respondents felt a withdrawal of this support at this point could truncate the progress of the PEI program. Government ownership in this regard would mean, for example, government taking leadership and funding its staff for regular supervision of RI sessions and surveillance. It would also mean dedicating staff to support the Fulani and other underserved populations for both polio and RI programs. This should be accompanied by other methods of staff motivation suggested by respondents such as promotion when due, reward for good performance, and provision of work infrastructure. A trained and motivated government workforce will address government´s human resource needs in the long term as opposed to NSTOP increasing its staff as recommended by many respondents. In addition, an increase in NSTOP staff without concurrent government ownership and capacity building of government officers will only worsen the issue of partner dependency that Nigeria has been struggling with for many health-related projects. On the other hand, the problem of partner dependency may also be related to the narrow focus of most partner projects such as a disease eradication goal, rather than projects that address the foundation of health system weakness. As such, partners may adopt strategies and tactics that are not conducive to government ownership in the long term.

There are some limitations to this assessment. The first is the design which largely involved description of program inputs and outcomes, and before-after comparison rather than using an external comparison group. An external comparison group is ideal to evaluate program as it generates the strongest evidence for attribution and may limit the impact of confounders [10,11]. This would not have been feasible in this setting, however, as NSTOP was a large-scale programmatic intervention to address specific public health needs. The program was implemented in a complex environment that could not be controlled nor extensively measured to capture all potential confounders. The NSTOP-supported LGAs were selected based on their high-risk status for polio transmission and it was impossible to identify similar LGAs that had no intervention, since the goal of the country PEI is to eliminate polio transmission. Secondly, from the interviews and focus groups, responder bias could have influenced the findings. We addressed this by interviewing a wide pool of stakeholders who had interacted with NSTOP either at management or operational levels. Another limitation is that the sampling frame for the assessment was restricted to accessible LGAs, so the findings can only be generalized to accessible LGAs in NSTOP-supported states. Specifically, some LGAs in Borno, Yobe, and Kaduna states were not included in the assessment because they were inaccessible due to insecurity. Also, the methodology was not designed to assess the impact of NSTOP ad hoc support, such as deployment of MSTs for polio campaigns. In the future, an impact assessment of the program would be beneficial as Nigeria gets closer to interrupting polio transmission. It should however be noted that generating credible evidence on the impact of NSTOP support will be always be a challenge based on all the issues discussed in this paper.

## Conclusion

The majority of immunization officers at the LGA and health facility level reported improvement in knowledge and skills from NSTOP trainings. In health facilities served by NSTOP there was improvement in the quality of RI supervision and RI microplanning, and decreased vaccine stock-outs. NSTOP program expenditures increased by 775% in FY 2013, 149% in FY 2014 and 26% in FY2015. Funding was provided by CDC, USAID, and BMGF, with CDC being the largest funder. The top drivers for program expenditures overall were capacity building for RI strengthening, RI data quality improvement, and deployment of MSTs for polio campaigns. Lastly, government and partner staff expressed concern that withdrawal of NSTOP officers at this time will likely have a negative effect on immunization activities at state and LGA levels unless government takes ownership of these activities.

In order to improve the future implementation of NSTOP, we propose recommendations in three main areas - capacity building, program monitoring, and transition planning. On capacity building, NSTOP should give highest priority to building skills and institutional capacity beyond merely knowledge acquisition. The focus of capacity building should be on developing the management skills of the LGA team. As the capacity of the LGA team improves, NSTOP should continue to strive to achieve improvements in provision of RI services as measured by vaccine coverage and programmatic indicators. On program monitoring, the monthly review of NSTOP program monitoring data should be strengthened with a monthly analysis of key indicators using a standardized dashboard. Another major recommendation in this area is to ensure routine tracking of program expenditures to promote accountability and transparency. On transition planning, it is recommended that NSLOs continue their current TORs on supporting the polio eradication and RI programs in their LGAs for a maximum period of five additional years. NSLOs should always conduct field visits with at least one government LGA team member to ensure transfer of skills and prudent use of logistic funds. No later than the last year of support to the LGA, NSLOs should use their time to develop and implement a transition plan for the LGA to fully take over NSTOP-supported activities.

### What is known about this topic

- Lack of government ownership constitutes a threat to the sustainability of interventions designed to improve quality of immunization services in Nigeria.

### What this study adds

- To sustain the impact of immunization interventions in Nigeria, priority should be given to building management skills and institutional capacity at local government level.
